# An Overview and Comparison of Traditional Motion Planning Based on Rapidly Exploring Random Trees

**DOI:** 10.3390/s25072067

**Published:** 2025-03-26

**Authors:** Yang Chu, Quanlin Chen, Xuefeng Yan

**Affiliations:** 1 School of Computer Science and Technology, Nanjing University of Aeronautics and Astronautics, Nanjing 211106, China; chuyang_716@nuaa.edu.cn; 2 State Key Laboratory for Novel Software Technology, Nanjing University, Nanjing 210023, China; quanlinchen@smail.nju.edu.cn

**Keywords:** kinodynamic planning, motion planning, sampling-based algorithms

## Abstract

Motion planning is a fundamental problem in robotics that involves determining feasible or optimal paths within finite time. While complete motion planning algorithms are guaranteed to converge to a path solution in finite time, they are proven to be computationally inefficient, making them unsuitable for most practical problems. Resolution-complete algorithms, on the other hand, ensure completeness only if the resolution parameter is sufficiently fine, but they suffer severely from the curse of dimensionality. In contrast, sampling-based algorithms, such as Rapidly Exploring Random Trees (RRT) and its variants, have gained the increasing attention of researchers due to their computational efficiency and effectiveness, particularly in high-dimensional problems. This review paper introduces RRT-based algorithms and provides an overview of their key methodological aspects.

## 1. Introduction

Motion planning is a well-known and fundamental problem in robotics that involves finding a (optimal) path for a robot from its initial position toward goal regions while avoiding collisions with any obstacles in its environment. While not the only fundamental problem of robotics, motion planning has garnered significant attention from researchers due to widespread applications across various fields, including robotics [1], computer animation [2], medical procedures [3], and manufacturing [4,5].

Many motion planning algorithms have been proposed, including complete, resolution complete, heuristic, potential field, and sampling-based algorithms. Each method offers distinct advantages and limitations in terms of space complexity, time complexity, and path optimization.

Complete algorithms primarily focus on geometric methods [6,7,8,9] that are guaranteed to find a path solution in finite time. However, both finding the shortest path and finding a feasible path under velocity constraints are proven to be NP-hard [10].

Resolution-complete algorithms include cell decomposition methods [11] and graph search methods [12,13,14]. They can guarantee completeness and optimality if fine resolution parameters are provided. However, their computation time and memory space grow exponentially with increasing dimensionality.

Heuristic algorithms consist of neural networks (NNs) and meta-heuristic optimization algorithms. On the one hand, NNs, with their advantages such as nonlinear mapping capabilities and learning ability, are employed in various aspects of navigation, including processing sensory data [15], obstacle avoidance, and path planning [16,17,18,19]. Additionally, end-to-end learning approaches for driving have gained significant attention in recent years, such as Deepdriving [20] and ChauffeurNet [21]. However, their disadvantages include slow training speeds, the need for large datasets, and a lack of traceability. On the other hand, meta-heuristic optimization algorithms offer high success rates and the ability to manage complex constraints. Notable examples include Brain Storm Optimization [22], Genetic Algorithm [23], Particle Swarm Optimization [24], and Ant Colony Optimization (ACO) [25]. However, they require considerable time to reach optimal solutions, especially for large-scale problems.

The Artificial Potential Field (APF) [26] is a well-known method usually used to handle unknown and dynamic environments [27]. However, APF suffers from the problem of local minima. To address this issue, APF is combined with various heuristics to achieve path optimality and safety, such as APF with random sampling [28], APF with ACO [29], and APF with GA [30].

Sampling-based algorithms have emerged as particularly prominent in recent years for two key reasons: (i) They avoid the explicit construction of obstacle configurations (which can be challenging in high-dimensional spaces). (ii) They allow the incorporation of differential constraints to generate dynamically feasible paths. Among sampling-based methods, Rapidly Exploring Random Tree (RRT) [31] and its asymptotically optimal variant (RRT* [32]) are widely recognized as foundational algorithms.

Despite the success of RRT*-based algorithms, there are still some major limitations [33]: (i) Their slow convergence rate to optimal solutions; (ii) Their significantly large memory requirements due to numerous iterations utilized to calculate the optimal path. Various improvements have been proposed to address these challenges. Some approaches focus on enhanced sampling strategies to replace pure uniform sampling, such as Informed-RRT* [34], RRT*-Smart [35], and CE-RRT* [36]. Other methods employ two directional trees with greedy connect heuristics to enhance the convergence rates of RRT and RRT*, including RRT-Connect [37], B-RRT* [38], and IB-RRT* [33].

Recent research has increasingly focused on extending sampling-based algorithms to handle dynamic systems. While RRT and RRT* excel at high-level path planning, they lack a low-level control synthesis. Several approaches attempt to bridge the gap by designing controllers to steer the system between two generated vertices, such as Kinodynamic-RRT* [39] and LQR-RRT* [40]. For environments with dynamical obstacles, CBF-RRT [41] incorporates control barrier functions (CBFs) into RRT to achieve dynamical obstacle avoidance. Similarly, CBF-RRT* [42] combines CBFs for safety with control Lyapunov functions (CLFs) to generate safe motion trajectories between vertices of RRT*.

This paper is structured as follows: Section 2 introduces fundamental components of RRT-based motion planning. Various RRT-based algorithms are introduced in Section 3. Section 4 introduces RRT-based algorithms for dynamic systems. Section 5 presents experimental results and analysis, and the conclusion is in Section 6.

## 2. Background

In this section, we introduce key local motion planning components commonly integrated into RRT-based algorithms. We focus on three fundamental approaches: linear quadratic regulators (LQRs), which optimize control inputs to minimize cost functions, control barrier functions (CBFs), which ensure safety constraints and obstacle avoidance, and control Lyapunov functions (CLFs), which guarantee stability and convergence properties.

### 2.1. System Dynamics

Consider robot dynamics modeled as affine control dynamics,

(1) $$\dot{x}=f_{\mathrm{cl}}\left(x,u\right):=f\left(x\right)+g\left(x\right)u,$$

with $f:R^{n}\to R^{n}$ and $g:R^{n}\to R^{n\times m}$ locally Lipschitz, the state $x\in X\subset R^{n}$, and the control input $u\in U\subset R^{m}$. The initial state is denoted as $x_{\mathrm{init}}$ and the goal region is denoted as $X_{\mathrm{goal}}\subset X$. $O_{i}\subset X$ represents the i-th obstacle region, where i=1,…,n. The obstacle-free set is defined as $X_{\mathrm{safe}}:=X-{⋃}_{i=1}^{n}O_{i}$.

### 2.2. Linear Quadratic Regulator

An LQR is a widely used optimal control strategy that minimizes a quadratic cost function. Consider a linear time-invariant system

$$\dot{x}=\mathrm{Ax}+\mathrm{Bu},$$

which is a special case of the general affine system (1) where f(x)=Ax,g(x)≡B. The LQR problem is to find control inputs u(t) that minimize the following quadratic cost criterion:

$$J_{\mathrm{LQR}}:=\int_{0}^{\infty }x{\left(t\right)}^{⊤}\mathrm{Qx}\left(t\right)+u{\left(t\right)}^{⊤}\mathrm{Ru}\left(t\right)dt,$$

where Q and R are positive-definite weighting matrices.

According to Theorem 10.1 in [43], if there exists a symmetric solution P to the following algebraic Riccati equation:

(2) $$A^{⊤}P+\mathrm{PA}+Q-{\mathrm{PBR}}^{-1}B^{⊤}P=0,$$

for which $A-{\mathrm{BR}}^{-1}B^{⊤}P=0$ is a stability matrix, then the optimal feedback control law is given by

$$u=-\mathrm{Kx},\;\mathrm{where}\;K:=R^{-1}B^{⊤}P,$$

with the optimal cost equal to $x{\left(0\right)}^{⊤}Px\left(0\right).$ This feedback law also stabilizes the closed-loop system.

In addition, LQR can be extended to handle nonlinear system dynamics through local linearization. More specifically, a local linearization can be made around an equilibrium point $\left(x^{\mathrm{eq}},u^{\mathrm{eq}}\right)$ using first-order Taylor expansion [43]:

$$\dot{\delta x}=\hat{A}\delta x+\hat{B}\delta u,$$

defined by the following Jacobian matrices:

$$\hat{A}:=\frac{\partial f_{\mathrm{cl}}\left(x^{\mathrm{eq}},u^{\mathrm{eq}}\right)}{\partial x},\;\hat{B}:=\frac{\partial f_{\mathrm{cl}}\left(x^{\mathrm{eq}},u^{\mathrm{eq}}\right)}{\partial u},$$

where $\delta x:=x-x^{\mathrm{eq}},\;\delta u:=u-u^{\mathrm{eq}}$, $\dot{\delta x}:=\dot{x}-\dot{x^{\mathrm{eq}}}$. Therefore, this local linearization allows the application of LQR techniques to nonlinear systems in the neighborhood of the equilibrium point.

### 2.3. Control Barrier Functions

CBFs are employed to generate control inputs that steer the system (1) towards an exploratory point while ensuring obstacle avoidance. Let the *safe set* C be defined as

C={x∈D:h(x)≥0},

for a continuously differentiable function $h:D\subset R^{n}\to R.$ The boundary and interior of C are given by

∂C={x∈D:h(x)=0}, Int(C)={x∈D:h(x)>0}.

The set C is called *forward invariant* for the system (1) if x(0)∈C implies x(t)∈C, ∀t>0. Intuitively, set invariance ensures that any trajectory starting within the invariant set will never leave it, thereby guaranteeing safety.

First, consider a model without control inputs, $\dot{x}=f_{\mathrm{cl}}\left(x\right)$. According to Nagumo’s Theorem [44], the necessary and sufficient conditions for set invariance are

$$\dot{h}\left(x\right)\geq 0\;\forall x\in \partial C.$$

Alternatively, another necessary and sufficient condition [45] is the existence of an extended class K function α such that

(3) $$\dot{h}\left(x\right)\geq -\alpha \left(h\left(x\right)\right),\;\forall x\in D.$$

The function *h*, which defines the forward invariant set, is called a *zeroing barrier function*.

Next, consider the system dynamics (1). The derivative of *h* becomes

$$\dot{h}\left(x\right)=L_{f}h\left(x\right)+L_{g}h\left(x\right)u.$$

Combining the above equation with Equation (3), for any point x∈D, the set of all controllers rendering the set C forward invariant is defined as

$$K_{\mathrm{zcbf}}\left(x\right):=\left\{u\in U:L_{f}h\left(x\right)+L_{g}h\left(x\right)u\geq -\alpha \left(h\left(x\right)\right)\right\}.$$

The function *h* is called a *zeroing control barrier function* (ZCBF) if $K_{\mathrm{zcbf}}\left(x\right)\neq \emptyset ,\;\forall x\in D$.

Finally, given a ZCBF *h*, safety can be ensured by modifying an existing controller in a minimal way. More specifically, given a feedback controller u=k(x) for the control system (1), it may happen that $k\left(x\right)\notin K_{\mathrm{zcbf}}\left(x\right)$ for some x∈D. To guarantee safety, one can determine the minimum perturbation to u by solving the following quadratic program (CBF-QP):

(4) $$\begin{array}{cc} u\left(x\right)=\underset{u\in R^{m}}{\arg\min} & \frac{1}{2}{∥u-k\left(x\right)∥}^{2}\\ s.t. & L_{f}h\left(x\right)+L_{g}h\left(x\right)u\geq -\alpha \left(h\left(x\right)\right). \end{array}$$

### 2.4. Control Lyapunov Functions

CLFs can be employed to generate control inputs that drive a system to a desired *equilibrium point* $\left(\bar{x},\bar{u}\right)$ that is, $f_{\mathrm{cl}}\left(\bar{x},\bar{u}\right)=0.$ Without loss of generality, the equilibrium point is typically assumed at the origin $(\bar{x}=0,\bar{u}=0).$ Additionally, it is typically assumed that the equilibrium point is stable and there exists an asymptotically stable neighborhood around it. More specifically, the equilibrium point is *stable* [46] if ∀ϵ>0∃δ>0,

∥x(0)∥<δ⇒∥x(t)∥<ϵ,∀t≥0.

Intuitively, the stability implies that a trajectory starting in a δ neighborhood of the origin will never leave the ϵ neighborhood. Moreover, a neighborhood S of the stable equilibrium point is called the region of *asymptotic stability* [46] if

$$∥x\left(0\right)∥\in S\Rightarrow \lim_{t\to \infty }x\left(t\right)=0.$$

Intuitively, the asymptotic stability implies that any point starting within S converges to the stable equilibrium point as *t* tends to infinity.

First, consider a model without control inputs, $\dot{x}=f_{\mathrm{cl}}\left(x\right)$. According to Theorem 2.26 in [44], a neighborhood S of origin is the region of asymptotic stability if there exists a locally Lipschitz-positive definite function Ψ such that

S⊂N[Ψ,v]:={x:Ψ(x)≤v} for some v>0,

and for all x∈N[Ψ,v],

(5) $$D^{+}\Psi \left(x\right)\leq -\phi \left(∥x\left(t\right)∥\right)\;\mathrm{for}\;\mathrm{some}\;K\;\mathrm{function}\;\phi ,$$

where $D^{+}$ is a Dini derivative. And this function Ψ is called the *Lyapunov function* inside S.

Next, consider the system dynamics (1). The Dini derivative of Ψ is given by

$$D^{+}\Psi \left(x\right)=L_{f}\Psi \left(x\right)+L_{g}\Psi \left(x\right)u.$$

Combining the above equation and Equation (5), the set of all stabilizing controllers for every point x∈S is defined by

$$K_{\mathrm{clf}}\left(x\right):=\left\{u\in U:L_{f}\Psi \left(x\right)+L_{g}\Psi \left(x\right)u\leq -\phi \left(∥x\left(t\right)∥\right)\right\}.$$

The function Ψ is called the *control Lyapunov function* (CLF) if $K_{\mathrm{clf}}\left(x\right)\neq \emptyset ,\;\forall x\in S$.

Finally, given a CLF Ψ and ZCBF *h*, one can steer the state x to another state $x^{\prime }$ while guaranteeing safety by solving a sequence of QPs. More specifically, suppose $\left(x^{\prime },\bar{u}\right)$ is the equilibrium point of the system. Define new variables $z\left(t\right):=x\left(t\right)-x^{\prime }$ and $v\left(t\right):=u\left(t\right)-\bar{u}$ through a translation to establish a new system whose equilibrium point is the origin. The initial state is set to $z\left(0\right)=x-x^{\prime }$. By restricting the control inputs $v\left(t\right)\in K_{\mathrm{clf}}$ for all t>0, one can guarantee z(t) converges to the origin. This constraint can then be incorporated into the CBF-QP, resulting in a new problem termed the CLF-CBF-QP:

(6) $$\begin{array}{cc} v\left(z\right)=\underset{v\in R^{m}}{\arg\min}\; & \frac{1}{2}{∥v-k\left(z\right)∥}^{2}\\ s.t.\; & L_{f}h\left(z\right)+L_{g}h\left(z\right)v\geq -\alpha \left(h\left(z\right)\right)\\ & L_{f}\Psi \left(z\right)+L_{g}\Psi \left(z\right)v\leq -\phi \left(∥z\left(t\right)∥\right). \end{array}$$

## 3. The RRT-Based Motion Planning

In this section, several incremental RRT-based motion planning algorithms are introduced.

### 3.1. The RRT Algorithm and Its Variations

The Rapidly Exploring Random Tree (RRT) is introduced as an efficient data structure and sampling scheme to explore efficiently nonconvex high-dimensional spaces by growing the search tree toward goal areas. The major components of the RRT are as follows:

- Sample: The procedure provides independent identically distributed samples from $X_{\mathrm{free}}$.
- Steer: Given two states $x,x^{\prime }\in X$, the Steer procedure returns a state $z\in X_{\mathrm{free}}$ that lies between x and $x^{\prime }$ while maintaining ∥z−x∥≤η, for a prespecified constant η>0. It is typically defined by
  $$\mathrm{Steer}\left(x,x^{\prime }\right)=\underset{z\in X_{\mathrm{free}},∥z-x∥\leq \eta }{\arg\min}∥z-x^{\prime }∥.$$
- Nearest node: Given a tree T=(V,E) with a set of nodes V and a set of edges E, the Nearest(V,x) procedure provides a node in V that is closest to x, i.e.,
  $$\mathrm{Nearest}\left(V,x\right)=\underset{x^{\prime }\in V}{\arg\min}∥x-x^{\prime }∥.$$
- Collision test: Given two points $x,x^{\prime }\in X_{\mathrm{free}}$, the procedure $\mathrm{ObstacleFree}(x,x^{\prime })$ returns True if and only if the line segment between x and $x^{\prime }$ lies entirely within $X_{\mathrm{free}}$, i.e.,
  $$\mathrm{ObstacleFree}\left(x,x^{\prime }\right)=\left\{\begin{array}{cc} \mathrm{True}, & \mathrm{if}\;\left[x,x^{\prime }\right]\subset X_{\mathrm{free}},\\ \mathrm{False}, & \mathrm{otherwise}. \end{array}\right.$$

The body of RRT algorithms is presented in Algorithm 1. The algorithm proceeds as follows. First, a state is sampled from the configuration space, denoted as $x_{\mathrm{samp}}$. Next, the Extend procedure grows the tree T towards $x_{\mathrm{samp}}$, as outlined in Algorithm 2. More specifically, the Nearest procedure selects the closest neighbor node $x_{\mathrm{nearest}}\in T$ to $x_{\mathrm{samp}}$. The Steer procedure steers the state from $x_{\mathrm{nearest}}$ to $x_{\mathrm{samp}}$, resulting in a new state $x_{\mathrm{new}}$. Finally, the state $x_{\mathrm{new}}$ and the edge $\left(x_{\mathrm{nearest}},x_{\mathrm{new}}\right)$ are added to T if the ObstacleFree procedure returns True.

Additionally, RRT-Connect, a variation of RRT, employs a two-RRT planner to achieve rapid convergence to a solution. More specifically, it constructs two trees, $T_{1}$ and $T_{2}$, starting from $x_{\mathrm{init}}$ and $x_{\mathrm{goal}}$, respectively. In each iteration, both trees are extended towards a random state $x_{\mathrm{samp}}$ simultaneously. The algorithm maintains both trees at all times until they connect and a solution is found.

Last but not least, these algorithms ensure *probabilistic completeness*, i.e., as the number of iterations approaches infinity, the probability of finding a solution (if one exists) approaches one (see Theorem 3 in [47]).
**Algorithm 1:** Body of RRT and RRT* algorithms
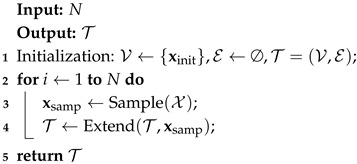

**Algorithm 2:** Extend_RRT_
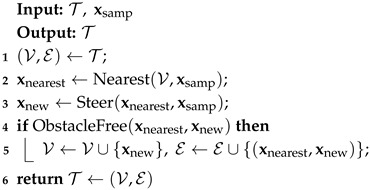


### 3.2. The RRT* Algorithm and Its Variations

Although RRT ensures probabilistic completeness, it does not guarantee optimality, i.e., it converges to a suboptimal solution with probability one (see Theorem 10 in [32]). To address this limitation, RRT* is introduced as an asymptotically optimal variant that asymptotically optimizes the existing tree to achieve *asymptotic optimality* properties, i.e., almost-sure convergence to an optimal solution. Several key components are added in RRT*:

- Near nodes: Given a tree T=(V,E) with a set of nodes V and a set of edges E, the Near(V,x) procedure utilizes a pre-defined distance ∥·∥ to identify a set of near nodes $X_{\mathrm{near}}$ in T that are close to x. This is typically defined as the set of all nodes within a closed ball of radius $r_{n}$ centered at x, i.e.,
  $$X_{\mathrm{near}}:=\left\{x^{\prime }\in V:∥x-x^{\prime }∥\leq r_{n}\right\},$$
  where the radius is defined by
  (7) $$r_{n}:=\gamma {\left(\frac{\log n}{n}\right)}^{1/d},$$
   *n* is the number of vertices in the tree, *d* is the dimension of the space, and γ is a constant.
- Choose Parent: The procedure tries to find collision-free paths between a selected node x and all its near nodes, and selects the near node that yields the lowest cost of x as the parent of x.
- Rewire: The procedure attempts to connect a selected node x with each node in its neighborhood $X_{\mathrm{near}}$. If the trajectory from x to the near node $x_{\mathrm{near}}$ results in a lower cost for $x_{\mathrm{near}}$, then x becomes the new parent of $x_{\mathrm{near}}$.

It is worth noting that the Rewire procedure guarantees the asymptotic optimality properties of RRT*.

The body of the RRT* algorithm is outlined in Algorithm 1, while the Extend procedure for the RRT* is detailed in Algorithm 3. The Extend procedure operates as follows. First, the Nearest procedure selects the closest neighbor node $x_{\mathrm{nearest}}\in T$ of $x_{\mathrm{samp}}$. Second, the Steer procedure steers the state from $x_{\mathrm{nearest}}$ to $x_{\mathrm{samp}}$, resulting in a new state $x_{\mathrm{new}}$. Third, the ChooseParent procedure identifies a potential parent node for $x_{\mathrm{new}}$. If a suitable parent node $x_{\mathrm{parent}}$ is found, then $x_{\mathrm{new}}$ and the edge $\left(x_{\mathrm{parent}},x_{\mathrm{new}}\right)$ are added to the tree T. Fourth, the Rewire procedure attempts to connect $x_{\mathrm{new}}$ with each node in its neighborhood $X_{\mathrm{near}}$. If the trajectory from $x_{\mathrm{new}}$ to the near node $x_{\mathrm{near}}$ results in a lower cost for $x_{\mathrm{near}}$, then $x_{\mathrm{new}}$ becomes the new parent of $x_{\mathrm{near}}$.

Although RRT* ensures asymptotic optimality, its convergence rate is relatively slow. More specifically, let f(x) be the cost of an optimal path from $x_{\mathrm{init}}$ to $x_{\mathrm{goal}}$ constrained to pass through x. The set of states that could improve the current solution is defined by

$$X_{f}:=\left\{x\in X:f\left(x\right)<c_{\mathrm{best}}\right\},$$

where $c_{\mathrm{best}}$ is the cost of the current solution. In RRT*, the probability of sampling states from $X_{f}$ becomes arbitrarily small for large subsets (see Remark 1 in [34]), leading to a slow convergence rate. Rapid convergence can be expected only if the probability of sampling states from $X_{f}$ increases.
**Algorithm 3:** Extend_RRT*_
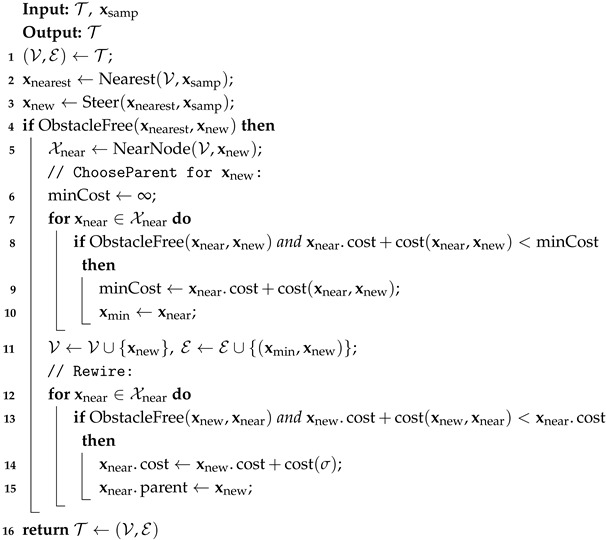


Several methods aim to enhance the probability of sampling states from $X_{f}$ through sample biasing. For instance, Informed-RRT* employs the heuristic $\hat{f}\left(x\right):=∥x_{\mathrm{init}}{-x∥}_{2}+{∥x-x_{\mathrm{goal}}∥}_{2}$ to estimate the unknown f(x). The set $X_{f}$ is then estimated by

$$X_{\hat{f}}:=\{x\in X:∥x_{\mathrm{init}}{-x∥}_{2}+∥x-x_{\mathrm{goal}}{∥}_{2}\leq c_{\mathrm{best}}\}.$$

The Sample procedure of Informed-RRT* is modified to generate uniformly distributed samples from the hyperellipsoid $X_{\hat{f}}$.

RRT*-Smart also implements biased sampling to accelerate the convergence rate. It tends to sample new states as close as possible to obstacles, inspired by visibility graph techniques, which optimize the existing path at turns.

Another approach is adaptive sampling, utilized by CE-RRT*, which applies the cross-entropy method to estimate the probability $P(X_{f})$. This estimator is then used to sample trajectories, resulting in a faster convergence rate.

Similar to RRT-Connect, there is also a bidirectional version of RRT* known as B-RRT*. B-RRT* employs a slight variation of the greedy RRT-Connect heuristic for connecting two trees. However, using two directional trees with a greedy connection heuristic does not guarantee asymptotic optimality.

## 4. The RRT-Based Motion Planning with Dynamics

It is challenging to apply RRT* to problems with dynamics due to the need for two domain-specific extension heuristics [40]: a distance metric and a steering procedure.

On the one hand, while the Euclidean distance is widely used in high-level path planning, it may not accurately reflect the dynamics of particular domains, especially in cases involving complex or underactuated dynamics. This can lead to poor exploration and slow convergence to optimal solutions.

On the other hand, a prevalent steering procedure in high-level path planning involves generating straight paths between vertices. However, steering safe trajectories between vertices in RRT* becomes challenging when considering low-level control synthesis.

In recent years, significant efforts have been made to adapt RRT-based algorithms to dynamic domains. This section will look at these methods that address the said challenges by leveraging either LQRs or CBFs.

### 4.1. The LQR-RRT* Algorithm

LQR-RRT* [48] is an extension of RRT* that integrates LQR into the Steer, Near, and Nearest procedures for optimal control synthesis. More specifically, given an equilibrium point x, cost(v,x) is defined as the LQR cost from any state v to x under the LQR policy. The cost function over the neighborhood of x can be expressed as

$${\cos t}_{x}\left(v\right)={\left(v-x\right)}^{⊤}P\left(v-x\right),$$

where P is a solution to Equation (2). Then, the three components of RRT* are modified as follows:

- LQRNearest: The LQRNearest(V,x) procedure identifies a node in V that is closest to x according to the cost function, i.e.,
  $$x_{\mathrm{nearest}}=\underset{v\in V}{\arg\min}{\cos t}_{x}\left(v\right).$$
- Near nodes: the LQRNear(V,x) procedure employs the cost function to find a set of near nodes $X_{\mathrm{near}}$ in T that are close to x, i.e.,
  $$X_{\mathrm{near}}:=\left\{v\in V:{\cos t}_{x}\left(v\right)\leq r_{n}\right\},$$
  where $r_{n}$ is consistent with Equation (7).
- Steer: Given two states, $x,x^{\prime }$, the LQR controller generates a sequence of optimal controls $\left\{u_{i}\right\}$ that steer a state trajectory from x to $x^{\prime }$.

### 4.2. The CBF-RRT Algorithm and Its Variations

CBF-RRT is an extension of RRT that incorporates CBFs into the steering function to ensure safety, eliminating the need for the Nearest or ObstacleFree procedures of RRT. The Steer procedure is modified as follows:

- Safe steer: Given a state $x_{0}$, a time horizon $t_{h}$, and a control reference k(x), the $\mathrm{SafeSteer}(x_{0},t_{h},k)$ procedure steers the state $x_{0}$ to an exploratory new state $x_{\mathrm{new}}$. Concretely, it generates a sequence of control inputs u(t) by solving a sequence of CBF-QPs (Equation (4)) that steers a collision-free trajectory to $x_{\mathrm{new}}$ at time $t_{0}+t_{h}$.

The overall algorithm of CBF-RRT is outlined in Algorithm 4.

Similar to CBF-RRT, CBF-RRT* incorporates CBFs into the RRT* algorithm to ensure safety. In addition to safety, RRT* requires the exact local motion planning because it needs to steer $x_{\mathrm{near}}$ to $x_{\mathrm{new}}$ in the ChooseParent procedure and steer $x_{\mathrm{new}}$ to $x_{\mathrm{near}}$ in the Rewire procedure. Consequently, CBF-RRT* implements the exact motion planning by solving a sequence of CLF-CBF-QPs (Equation (6)). The state trajectory from $x_{\mathrm{new}}$ to $x_{\mathrm{near}}$ can be also utilized to evaluate the cost between the two states, offering greater precision than the LQR cost-to-go function used in LQR-RRT*.

**Algorithm 4:** CBF-RRT

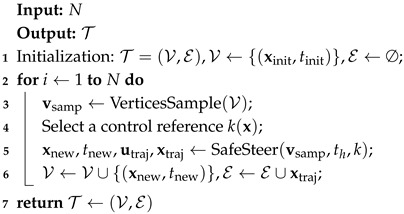



LQR-CBF-RRT* [48], an incremental version of CBF-RRT*, not only incorporates CBFs into the steering function for safety but also employs LQR for optimal control synthesis. Additionally, it proposes truncating the state trajectory that violates CBF constraints to reduce the burden of solving the QPs, leading to a new Steer procedure:

- LQR-CBF-Steer: Given two states $x,x^{\prime }$, the LQR controller generates a sequence of optimal controls $\left\{u_{i}\right\}$ that steer a state trajectory from x to $x^{\prime }$. The CBF constraints are checked to ensure that the generated trajectory is collision-free. If none of the CBF constraints are violated, then $x^{\prime }$ is added to the tree. Otherwise, the trajectory is truncated so that the end state $x_{\mathrm{new}}$ is safe, and the end state $x_{\mathrm{new}}$ is then added to the tree.

## 5. Experimental Results

In this section, we conduct an experimental study to compare the performance of several baseline methods.

### 5.1. Path Planning

We evaluated RRT, RRT*, RRT-Connect, Informed-RRT*, and RRT*-Smart on two examples for planning the motions of rigid objects in 2D. The implementations of RRT, RRT*, RRT-Connect, RRT*-Smart, and Informed-RRT* were based on the reference code from the package https://github.com/zhm-real/PathPlanning (accessed on 10 December 2024). The hyperparameters for these methods were set as follows: step_len=1, goal_sample_rate=0.1, and search_radius=12 (applied to RRT*-based methods only).

**Figure 1:**
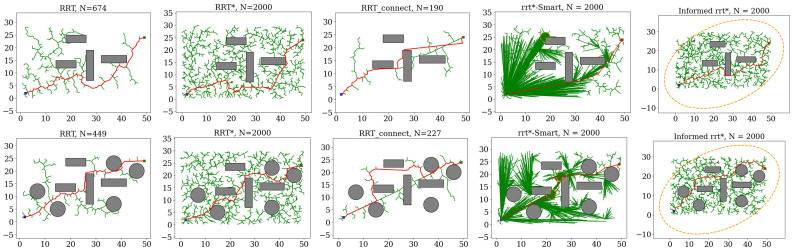
The evolution of baseline methods on two examples. In the visual representation, green edges represent sampled trajectories, the red trajectory highlights the final solution achieved after 1000 iterations, and gray areas represent obstacles.

Figure 1 illustrates the evolution of baseline methods across both examples. The comparison between RRT and RRT* demonstrates that RRT* can asymptotically optimize the existing path but its convergence rate is notably slow. The comparison between RRT and RRT-Connect demonstrates that the greedy behavior of RRT-Connect improves both the convergence rate and final solution quality of RRT. With the direct sampling of an ellipsoidal subset, Informed-RRT* improves the convergence rate and final solution quality compared to RRT*. Additionally, with intelligent sampling and path optimization, RRT*-Smart produces significantly shorter paths than RRT*.

**Figure 2:**
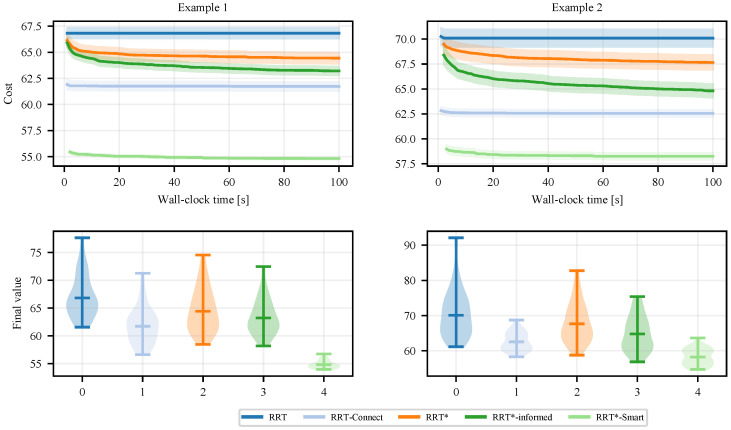
(**Top row**) Costs are plotted against the running time showing the convergence rate of baseline methods, averaged over 50 runs. (**Bottom row**) The distribution over the final costs over 50 runs.

Figure 2 presents the convergence pattern of the costs associated with baseline methods on these two examples. RRT*-Smart demonstrates the fastest convergence rate and the highest final solution quality among all baseline methods. This highlights the effectiveness of the intelligent sampling and path optimization techniques introduced by RRT*-Smart. Notably, RRT-Connect also performs exceptionally well, as its greedy connect heuristic significantly outperforms both RRT* and Informed-RRT*. Additionally, Informed-RRT* shows improvements in both convergence rate and final solution quality compared to RRT*.

### 5.2. Double Integrator Model

Following [48], we considered a double integrator model with linear dynamics,

$$\ddot{x_{1}}=u_{1},\;\ddot{x_{3}}=u_{2}$$

with the state $x=[x_{1},x_{2},x_{3},x_{4}]$ where $\left[x_{1},x_{3}\right]$ denotes the position and $\left[x_{2},x_{4}\right]$ denotes the velocity. The control input $u=[u_{1},u_{2}]$ consists of acceleration.

We present an experimental performance comparison between LQR-RRT* and LQR-CBF-RRT*. The LQR-RRT* and LQR-CBF-RRT* used reference implementations from the package https://github.com/mingyucai/LQR_CBF_rrtStar (accessed on 10 December 2024). The hyperparameters for these methods were set as follows: step_len=10, goal_sample_rate=0.1, and search_radius=20.

**Figure 3:**
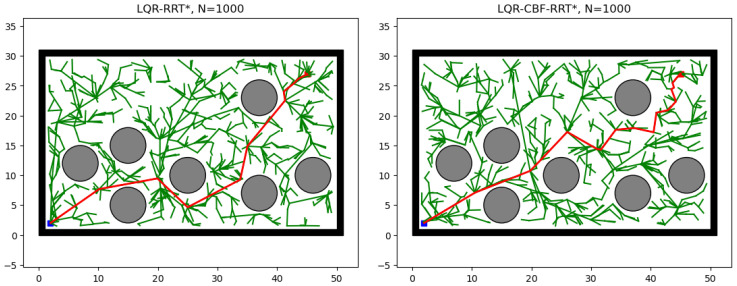
The evolution of LQR-RRT* and LQR-CBF-RRT* on the double integrator model. In the visual representation, green edges represent sampled trajectories, the red trajectory highlights the final solution achieved after 1000 iterations, and gray areas represent obstacles.

**Figure 4:**
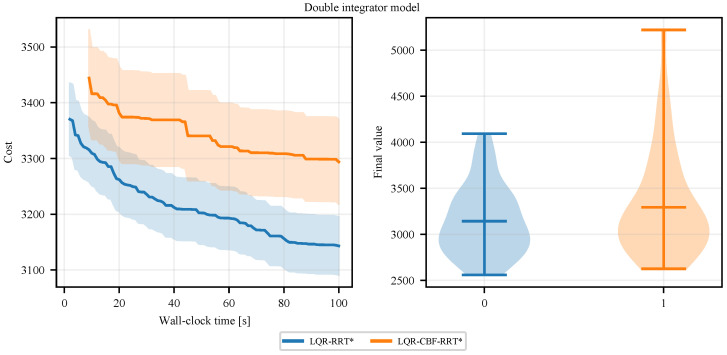
(**Left**) Costs are plotted against the running time showing the convergence rate of LQR-RRT* and LQR-CBF-RRT*, averaged over 50 runs. (**Right**) The distribution over the final costs over 50 runs.

Figure 3 shows the evolution of these two methods on the double integrator model. It can be observed that, given the same number of iterations, LQR-CBF-RRT* achieves a higher-quality final solution compared to LQR-RRT*. However, as is shown in Figure 4, LQR-RRT* achieves a faster convergence rate with respect to running time than LQR-CBF-RRT*. This difference may be attributed to the increased time complexity introduced by using CBFs in LQR-CBF-RRT*.

## 6. Conclusions

In this paper, we have summarized the current state of RRT-based algorithms and conducted an experimental study to compare the performance of several baseline methods. Our results confirm a good performance of the greedy connection heuristic introduced by RRT-Connect, the intelligent sampling and path optimization introduced by RRT*-Smart, and the effectiveness of LQR-RRT*. However, the field still has much to explore. There are undoubtedly many new challenges, discoveries, and insights awaiting further investigation in the future.

## Data Availability

Data are contained within the article.
